# Author Correction: Deep learning on fundus images detects glaucoma beyond the optic disc

**DOI:** 10.1038/s41598-023-48939-z

**Published:** 2023-12-05

**Authors:** Ruben Hemelings, Bart Elen, João Barbosa-Breda, Matthew B. Blaschko, Patrick De Boever, Ingeborg Stalmans

**Affiliations:** 1 Research Group Ophthalmology, Department of Neurosciences, KU Leuven, Herestraat 49, 3000 Leuven, Belgium; 2 Ophthalmology Department, UZ Leuven, Herestraat 49, 3000 Leuven, Belgium; 3 Cardiovascular R&D Center, Faculty of Medicine of the University of Porto, Alameda Prof. Hernâni Monteiro, 4200-319 Porto, Portugal; 4 Department of Ophthalmology, Centro Hospitalar E Universitário São João, Alameda Prof. Hernâni Monteiro, 4200-319 Porto, Portugal; 5 ESAT-PSI, KU Leuven, Kasteelpark Arenberg 10, 3001 Leuven, Belgium; 6 Hasselt University, Agoralaan building D, 3590 Diepenbeek, Belgium; 7 Department of Biology, University of Antwerp, 2610 Wilrijk, Belgium; 8 Flemish Institute for Technological Research (VITO), Boeretang 200, 2400 Mol, Belgium

Correction to: *Scientific Reports* 10.1038/s41598-021-99605-1, published online 13 October 2021

The Code Availability section in the original version of this Article was omitted. It now appears as below:

“The standard ResNet-50 code used in this study is available at https://keras.io/api/applications/resnet/#resnet50-function. From the 64 models developed for glaucoma detection or VCDR regression, 62 are available upon academically reasonable request (e-mail: ingeborg.stalmans@kuleuven.be). Two models (glaucoma detection and VCDR regression without any cropping), in addition to the optic disc localization model, were transferred to Mona.health. Interested parties are invited to reach out to Mona.health (e-mail: info@mona.health). All of the code was developed and carried out in a kernel with Python version 3.6 on a Windows 10 machine.”

Furthermore, the Competing Interests section was incorrect.

“The authors declare no competing interests.”

now reads:

“No outside entities have been involved in the study design, in the collection, analysis and interpretation of data, in the writing of the manuscript, nor in the decision to submit the manuscript for publication. I.S. is co-founder, shareholder, and consultant of Mona.health, a KU Leuven / VITO spin-off to which three of the described models were transferred. Under their terms of employment at KU Leuven, R.H. and M.B.B. are entitled to stock options in Mona.health. The study design was conceptualized in light of the PhD thesis of R.H., prior to the model transfer. The KU Leuven investigators have a free research licence to the three transferred models.”

Additionally, the Acknowledgements section contained an error.

“The first author is jointly supported by the Research Group Ophthalmology, KU Leuven and VITO NV. This research received funding from the Flemish Government under the “Onderzoeksprogramma Artificiële Intelligentie (AI) Vlaanderen” programme. No outside entities have been involved in the study design, in the collection, analysis and interpretation of data, in the writing of the manuscript, nor in the decision to submit the manuscript for publication. Thus, the authors declare that there are no conflicts of interest in this work.”

now reads:

“The first author is jointly supported by the Research Group Ophthalmology, KU Leuven and VITO NV. This research received funding from the Flemish Government under the “Onderzoeksprogramma Artificiële Intelligentie (AI) Vlaanderen” programme. No outside entities have been involved in the study design, in the collection, analysis and interpretation of data, in the writing of the manuscript, nor in the decision to submit the manuscript for publication.”

The original Article has been corrected.

